# Long noncoding RNAs and metabolic memory associated with continued progression of diabetic retinopathy

**DOI:** 10.1111/1753-0407.70009

**Published:** 2024-11-18

**Authors:** Jay Kumar, Pooja Malaviya, Renu A. Kowluru

**Affiliations:** ^1^ Ophthalmology, Visual and Anatomical Sciences Wayne State University Detroit Michigan USA

**Keywords:** diabetic retinopathy, long noncoding RNA, metabolic memory

## Abstract

Progression of diabetic retinopathy resists arrest even after institution of intensive glycemic control, suggesting a “metabolic memory” phenomenon, but the mechanism responsible for this phenomenon is still elusive. Gene expression and biological processes can also be regulated by long noncoding RNAs (LncRNAs), the RNAs with >200 nucleotides and no open reading frame for translation, and several LncRNAs are aberrantly expressed in diabetes. Our aim was to identify retinal LncRNAs that fail to reverse after termination of hyperglycemia. Microarray analysis was performed on retinal RNA from streptozotocin‐induced diabetic rats in poor glycemic control for 8 months, followed by in good glycemic control (blood glucose >400 mg/dL), or for 4 months, with four additional months of good glycemic control (blood glucose <150 mg/dL). Differentially expressed LncRNAs and mRNAs were identified through Volcano filtering, and their functions were predicted using gene ontology and pathway enrichment analyses. Compared with age‐matched normal rats, rats in continuous poor glycemic control had >1479 differentially expressed LncRNAs (710 downregulated, 769 upregulated), and among those, 511 common LncRNAs had similar expression in Diab and Rev groups (139 downregulated, 372 upregulated). Gene Ontology/pathway analysis identified limited LncRNAs in biological processes, but analysis based on biological processes/molecular function revealed >350 genes with similar expression in Diab and Rev groups; these genes were mainly associated with stress response, cell death, mitochondrial damage and cytokine production. Thus, identifying retinal LncRNAs and their gene targets that do not benefit from termination of hyperglycemia have potential to serve as therapeutic targets to slow down the progression of diabetic retinopathy.

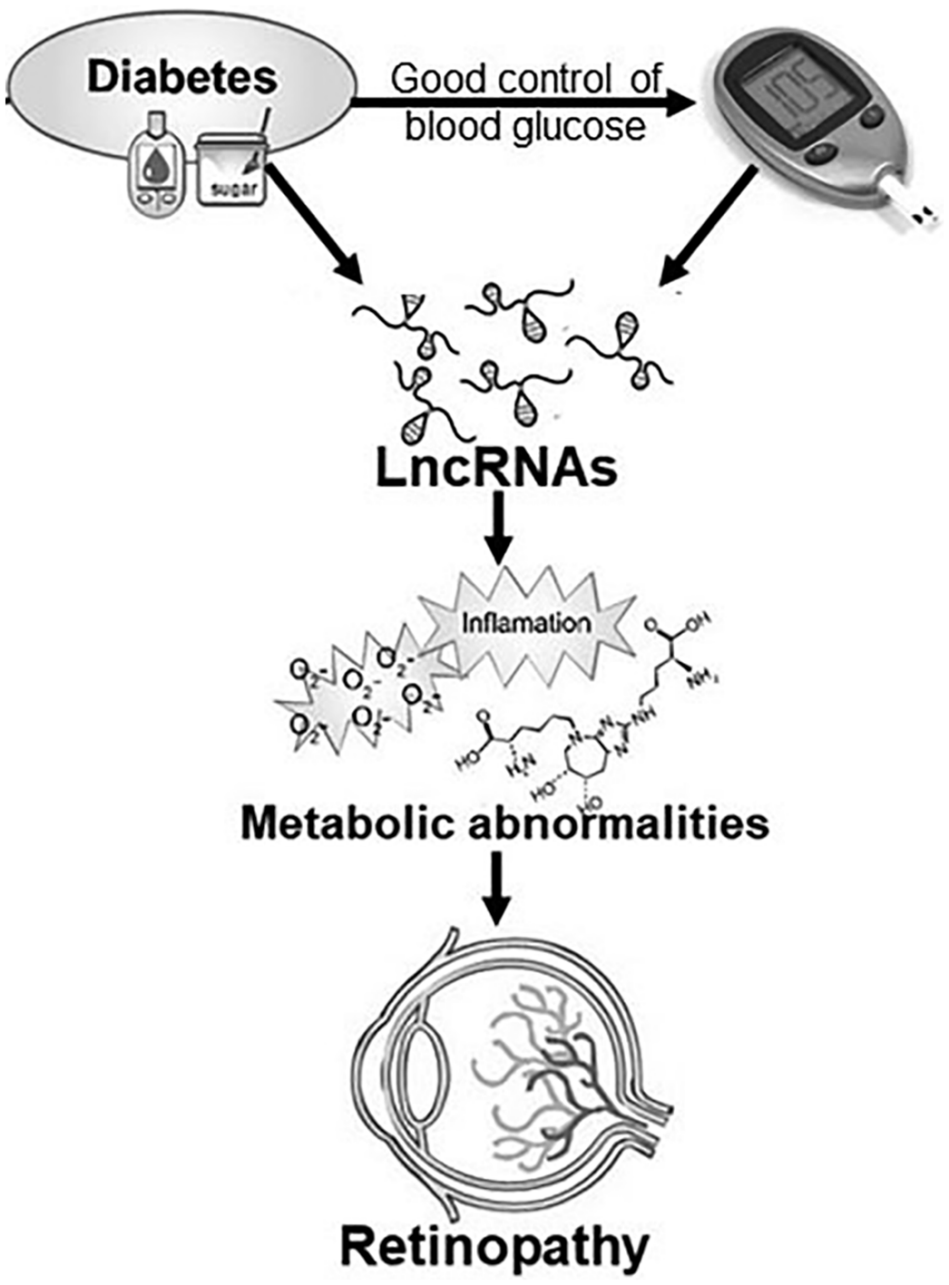

## Graphical abstract

Highlights

- Hyperglycemia results in aberrant expression of several LncRNAs (RNAs with >200 nucleotides), leading to altered expression of many genes and metabolic abnormalities implicated in the development of diabetic retinopathy.
- However, reversal of hyperglycemia by normoglycemia does not provide any benefit to these aberrantly expressed LncRNAs, and the retinopathy continues to progress.

## 1 INTRODUCTION

Pivotal Diabetes Control and Complications Trial (DCCT) and the follow‐up Epidemiology of Diabetes Interventions and Complications (EDIC) study have shown that the clinical features of retinopathy in the conventional glycemic control patients continue to develop long after hyperglycemia termination.^1^ These trials have also documented that maintenance of an intensive control during the early stages of diabetes is critical because the benefits of such control persist beyond the period of its institution. But, the prior poor glycemic history also imprints its effects on diabetic retinopathy; even after achieving and maintaining intensive control for many years, the damage instilled by the prior poor glycemic control becomes difficult to undo.^2^ Retinopathy incidence among the patients with conventional glycemic control is higher even 26 years after DCCT termination, compared with those in intensive glycemic control,^3^ and the increased risk of diabetic complications including retinopathy has continued in the conventional glycemic control group despite reverting to better glycemic control, suggesting a “metabolic memory” phenomenon. However, the molecular mechanism of metabolic memory phenomenon remains elusive.

Metabolic memory is successfully duplicated in the experimental models of diabetic retinopathy; chemically induced dog model of diabetic retinopathy has shown no signs of retinal histopathology during their 30 months of poor glycemic control, but at the end of 30 months of good glycemic control, which has followed the 30 months of poor glycemic control, histopathology is clearly detectable in their retina.^4^ Similarly, in streptozotocin‐induced diabetic rats, histopathology continues to develop even after maintenance of good glycemic control, which has followed a period of poor glycemic control. However, if good glycemic control is instituted soon after induction of diabetes in rats, the retina is protected from such damage, clearly suggesting that the submicroscopic processes, which begin during the initial period of poor glycemic control, fail to halt.^5^, ^6^ Metabolic memory phenomenon can also be duplicated in isolated retinal capillary cells incubated in varying glucose concentrations.^7^, ^8^ The memory phenomenon is complex with many metabolic abnormalities and epigenetic modifications, initiated by the prior glycemic control, continue to damage the retinal vasculature,^6^, ^8^, ^9^, ^10^, ^11^, ^12^ making the exact mechanism(s) elusive.

Recent sequencing technologies have revealed that despite ~85% of the human genome being actively transcribed, only ~2% encodes proteins, and the noncoding RNAs are approximately four times more than the coding RNA sequences.^13^ These noncoding RNAs are not totally unfunctional; they can regulate gene expression at the transcriptional and posttranscriptional levels and are shown to have regulatory roles in many biological processes.^14^ Among these, long noncoding RNAs (LncRNAs) are the nonprotein coding sequence transcripts with >200 nucleotides but have no open reading frame for translation.^15^ Unlike association of microRNAs (miRNAs) with gene regulation,^16^ LncRNAs field is in its early stages and their exact number in the human genome is still not clear; one recent transcriptomic/bioinformatic study has put their numbers in the tens of thousands.^17^ Long noncoding RNAs serve many key regulatory roles including regulating the activity or localization of proteins and serving as organizational frameworks of subcellular structures. They can also be processed to yield small RNAs or can modulate processing of other RNAs.^14^, ^15^, ^17^ LncRNAs have been implicated in the diabetes and its complications, including in the development of diabetic retinopathy.^18^, ^19^, ^20^, ^21^ Several retinal LncRNAs including LncRNAs *MALAT1*, *MIAT1*, *ANRIL*, and *CytB* are aberrantly expressed in diabetes and are associated with the metabolic abnormalities implicated in the development of diabetic retinopathy including inflammation, oxidative stress and mitochondrial damage.^20^, ^21^, ^22^, ^23^ However, it is unclear if LncRNAs have any role in the metabolic memory phenomenon associated with the progression of diabetic retinopathy.

Our aim was to identify aberrantly expressed LncRNAs that fail to reverse back after hyperglycemia is terminated. Using experimental models of diabetic retinopathy‐ metabolic memory, LncRNA array was performed in the retina of rats in poor glycemic control for 8 months, or in poor control for 4 months, followed by good glycemic control for four additional months.

## 2 METHODS

### 2.1 Rats

Wistar rats (male, ~200 g body weight), made diabetic by streptozotocin (55 mg/kg body weight, intraperotoneally), either remained in poor glycemic control (blood glucose >400 mg/dL; Diab group) throughout the 8‐month duration (Diab group) or were maintained in poor control for 4 months, followed by good glycemic control (blood glucose<150 mg/dL) for 4 months (Rev group). Good glycemic control was maintained by administering insulin two times a day (total 5–7 IU/day).^5^, ^6^, ^9^ Age‐matched, normal, nondiabetic rats were used as controls (Norm group); each group had seven to nine animals, and they were sacrificed by carbon dioxide inhalation. Retinal RNA was isolated from four different animals in each group (Norm, Diab, and Rev). The experimental protocols were approved by Wayne State University's Animal Care and Use Committee, and the treatment of the animals followed the guidelines of the Association for Research in Vision and Ophthalmology Resolution with the Use of Animals in Research.

### 2.2 Retinal histopathology

The whole retina (isolated from the formalin‐fixed eyes) was incubated with 3% crude trypsin (Invitrogen‐Gibco, Grand Island, NY, USA) containing 200 mol/L sodium fluoride for 60–70 min at 37°C. After gently brushing away the neuroretinal tissue under a microscope, the clean vasculature was stained with periodic acid‐Schiff–hematoxylin, and acellular capillaries were counted by light microscopy.^24^

### 2.3 Isolation of RNA and its quality check

Total RNA was isolated using RNeasy Micro Kit (Qiagen, Valencia, CA, USA), following the manufacturer's protocol and was quantified by NanoDrop ND‐1000 spectrophotometer. The purity and the quality of the RNA were assessed quantifying the absorbance ratio of the values at 260 and 280 nm, and by standard denaturing agarose gel electrophoresis.^25^ Samples with 260:280 nm ratio of 1.8 or higher were further processed for microarray analysis.

### 2.4 Array hybridization and data analysis

Briefly, RNA (4 rats/group) was amplified and transcribed into fluorescent complimentary RNA (cRNA) along the entire length of the transcripts without 3′ bias utilizing a random priming method (Arraystar Flash RNA Labeling Kit, Arraystar, Rockville, MD). The labeled cRNA was purified and the concentrations and specific activity of the labeled cRNAs (pmol Cy3/μg cRNA) were measured using NanoDrop ND‐1000 spectrophotometer. Labeled cRNA (1 μg) was fragmented in the fragmentation buffer (1 μL 25×) containing the blocking agent (5 μL 10×), and heated at 60°C for 30 min. The samples were diluted using 25 μL GE Hybridization buffer (2×), and the hybridization mixture (50 μL) was then transferred onto the gasket slide and assembled to the microarray slide. The labeled cRNA was hybridized in an Agilent hybridization oven at 65°C for 17 h onto the Rat LncRNA Array v3.0 (4 × 44 K, Arraystar); this allowed assessment of both LncRNA and messenger RNA (mRNA) on the same chip. (Please note: Rat LncRNA Array v3.0 can detect ~10 333 LncRNAs and 28 287 coding transcripts). Hybridized samples were washed, fixed, and scanned using Agilent DNA Microarray Scanner G2505C, and the acquired images were analyzed by Agilent Feature Extraction software (version 11.0.1.1).

Quantile normalization and subsequent data processing was performed using the GeneSpring GX v12.1 software package (Agilent Technologies, Santa Clara, CA, USA). Values for the raw microarray signal intensity were normalized across the arrays as normalized intensities for the comparison of expression levels, and LncRNAs and mRNAs, at least three out of twelve samples with flags in Present or Marginal (“All Targets Value”), were chosen for further analysis. Quality assessment of the microarray array data was done by plotting box plots to conveniently visualize the distributions of a dataset and Array QC reports, which included the QC metrics and statistics to evaluate the reproducibility and reliability of microarray data (data not included).

Differentially expressed LncRNAs and mRNAs with statistical significance, compared with normal control, were identified through fold change filtering between the two groups, and LncRNAs and mRNAs with statistically significant differences between the Norm and Diab, or the Norm and Rev groups, were identified through Volcano filtering by displaying fold change on the *x*‐axis and statistical significance on the *y*‐axis (represented as −log10 *p*‐values). Hierarchical cluster analysis of the top 50 downregulated and upregulated LncRNAs was performed using Heatmapper web server (http://www.heatmapper.ca/expression/).^26^

A group of downregulated and upregulated LncRNAs were validated by quantitative real‐time polymerase chain reaction (qRT‐PCR) and by semiquantitative PCR. TRIzol‐isolated RNA (2 μg) was used to synthesize cDNA using High‐Capacity cDNA Reverse Transcription Kit (Applied Biosystems, Waltham, MA, USA). qRT‐PCR was performed in triplicate in a 7500 Fast Real‐Time PCR System (Applied Biosystems) using the SYBR™ Green PCR Master Mix (Applied Biosystems); Table 1 lists the primer sequences used to quantify LncRNA transcripts. The transcript expression was normalized by the candidate reference gene (β‐actin), and the fold change was calculated using the 2^−ΔΔCt^ method.^22^, ^23^, ^27^

**TABLE 1:** Primer sequence.

	Forward (5′‐3′)	Reverse (5′‐3′)
LncRNA		
*LOC100362486*	TCGCACCAGCAAGATGAGTT	ACTGCTTGGAACGGGCATTA
*Ankef1*	GGCAGACATGTTGGTCAAGC	GGCTACTATCAAGTAGGCAGCA
*Csnk1d*	CCTCATCTGATCTCACGGGC	CAGTAGGTGGTACGTCGTGG
*Ankle1*	CTTGCCTGTTGGATGCCCTA	AGCAGGTATACGCCCAATCG
*Gpr61*	CAACTTCTGGGTAGGCATTGGA	GGAGGCCAGTCCTTGTCTCAG
*Nprl3*	GCCTAATGGCATCACCCAGT	ACAGATGTCCTGGAAGGGGA
*Fancg*	TCGAGTGGCTGCTCTGATTA	AATCTTTTCAGAGTCTGGGCAT
*LOC100361265*	CATGATCAGGAGAGGGACGC	TTCTTGGGGTCATCCAAGCC
*Ccdc70*	TGATGTGACAATGTCCGCCT	CTGGAATGGTGGATGGCTGT
*Tchhl1*	GTCGAGGCTCCTGAGAGGTA	ATGTGGCTGAAGGAAGTCCC
*Efcab1*	CAGCAAAAACTACCGTGGGC	TGTGAGGAAGAATGCTGGTCT
*Slc7a6*	CACAGCCACCAAAGACCAGT	ACTACAAAGAGCAATGAGAGAGCA
*Prpf4b*	CTATCCGGATTCTTAGCGCGG	TTGAACTTCCCGACTTCTCCG
*Srsf2*	GGGCGTGTATTGGAGCAGAT	GCTACACAACTGCGCCTTTT
*LOC100911891*	CAGTGGTGGTTGTAACGGGA	CGCGAAATTTTGCTCCAGGT
*LOC100363520*	GCATTGTAGCTGAGACCCCT	CCAGCCCCTACTCCTCTACAA
*Ikbke*	AGAGAAGGCCTGTACCTCCC	TTCCCGGTCAGGTACCGTAG
*MALAT1*	AAGATGAGGGTGTTTACG	AAGCCTTCTGCCTTAGTT
*NEAT1*	GTCAGACACTGGATGGTGGG	GCAAGACAAGGTGTGGCAAG
*HOTAIR*	TTGGGACTAAAGGGAGCCGA	CAGTTCTCTCGGCTGGGTG
*Meg3*	GGACATCGAGGGACAAGCAA	GAGCAAAGGTTGAGGGGTCA
*CARL*	GTTAGTGGTGAGACCCGGTG	TCTCTGGCATCCATTTGTGCT
*CytB*	CGGAATGTTAGGCTGCGTTG	CCATTCTACGCTCCATCCCC
*ND5*	TGGGTGCAAATGTGGAGGAA	TGTGCTCTCACCCAAAACGA
*ND6*	ATCCCCGCAAACAATGACCA	TTGGGGTTGCGGCTATTTAT
mRNA		
*GPx2*	AAAGACAAGCTGCCCTACCC	TTCCAGGACACATCTGAGCG
*Opn5*	CGTTGACTGCGGTGACGATA	GGAATGATGCACTTCCCAAGC
*Fmo4*	AGCAACGGGCTTTTGTGTTG	TTTCGGTCTCAGAGCCTTGTC
*Cox19*	GTCGACCGCAATGAACTTCG	TCTTGTCGCGGAGACACTTC
*Fmc1*	CAAGGACAGGAAAATGGCGG	GTGCTCGGAAAGCCTTAACC
*Lox*	CAGCTACCTGGTGCCTGAAT	AGGTACTGCTTCATCCTTTGGG
*Emg1*	CTTCCGTACAGGTATCGCCA	CTGGTCGCAGCCATCTTGTA
*Mtch1*	CGACCAGAATCCAGGTTCCC	ACGAGCAGGAAGGGATAGGT
*Bag3*	CTGACTGCTCATCCTCGTCC	CCGGGTGATGTTCTGCTCAT
*YWHAB*	ACCACAAAAAGGTCCCGTGT	CTCTCCTTCAGCTAGGGGGT
*β‐Actin*	CCTCTATGCCAACACAGTGC	CATCGTACTCCTGCTTGCTG

Abbreviation: LncRNA, long noncoding RNA.

For semiquantitative PCR, 3 μL cDNA was mixed with 10 μL GoTaq® Green Master Mix (Cat. No. M712B, Promega, Madison, Wisconsin, USA), 5 μL nuclease‐free water, and 1 μL each of forward and reverse primers. The thermal cycling conditions included 95°C for 5 min, 35 cycles of 95°C for 30 s, 60°C for 1 min, 72°C for 1 min, and the final extension at 72°C for 5 min. PCR products were analyzed on a 2% agarose gel, and the band intensity of the products was quantified using Syngene InGenius3 Gel Imaging System (Fisher Scientific, Waltham, MA, USA). Relative expression of LncRNAs was calculated by normalizing the band intensities with that of β‐actin in each sample.

### 2.5 Gene Ontology and Kyoto Encyclopedia of Genes and Genomes pathway analyses

Gene ontology (GO), a bioinformatics tool to access information about gene product function using ontologies to represent biological knowledge, and pathway enrichment analyses, were performed to predict the functions of differentially expressed LncRNA and genes. GO analysis (http://geneontology.org/) was performed based on three integrated domains, GO‐biological process, molecular function and cellular component,^25^, ^28^ and the GOs common between the Norm and Diab, Norm and Rev, and Diab and Rev groups were identified.

Biological pathways associated with the differentially expressed mRNAs were determined using Kyoto Encyclopedia of Genes and Genomes (KEGG: www.genome.jp/kegg) enrichment analysis, a bioinformatics database which provides information on metabolic pathways, regulatory networks, and functional annotations of genes.^29^ The common KEGG pathways between the Norm and Diab, Norm and Rev, and Diab and Rev groups were identified. Hierarchical Clustering was performed to show the differentially expressed LncRNAs and mRNAs patterns among the samples.

### 2.6 Coding‐noncoding (mRNA–LncRNA) coexpression network

Differentially expressed LncRNAs and their targeted coding genes were identified by a coexpression network of mRNA–LncRNA using Cytoscape software (coding‐noncoding genes, CNC network, https://cytoscape.org/) to characterize correlation (negative and positive) between LncRNA and mRNA. Pearson correlation coefficient was calculated for each pair of differentially expressed mRNA and LncRNA, and coefficients equal or >0.995 were applied for the CNC network.^30^ The number of directly linked neighbors was used as the “degree”, and was considered as the most important measure of the centrality of a gene within a network and indicated its relative importance.

To further confirm LncRNA–mRNA coexpression, eight LncRNAs were selected from the list identified by microarray on the basis of their characterization and availability in the literature. Pearson correlation coefficient was calculated for each pair of differentially expressed mRNA and LncRNA, and coefficients equal or >0.995 were used for the CNC network. These LncRNAs were then analyzed for their coexpression with the mRNAs to build the coexpression network using Cytoscape software, and among those 10 mRNAs showing correlation with at least one of the LncRNAs were then selected for further analysis by qRT‐PCR using gene specific primers and β‐actin as a housekeeping gene (Table 1).

### 2.7 Statistical analysis

Statistical analyses were performed using Graph Pad Prism (San Diego, CA), and the results were expressed as mean ± SD. Significance of variance was analyzed using one‐way ANOVA, followed by post‐hoc analysis, and a *p* value <0.05 was considered as statistically significant.

## 3 RESULTS

### 3.1 Severity of hyperglycemia in rats and retinal histopathology

**FIGURE 1:**
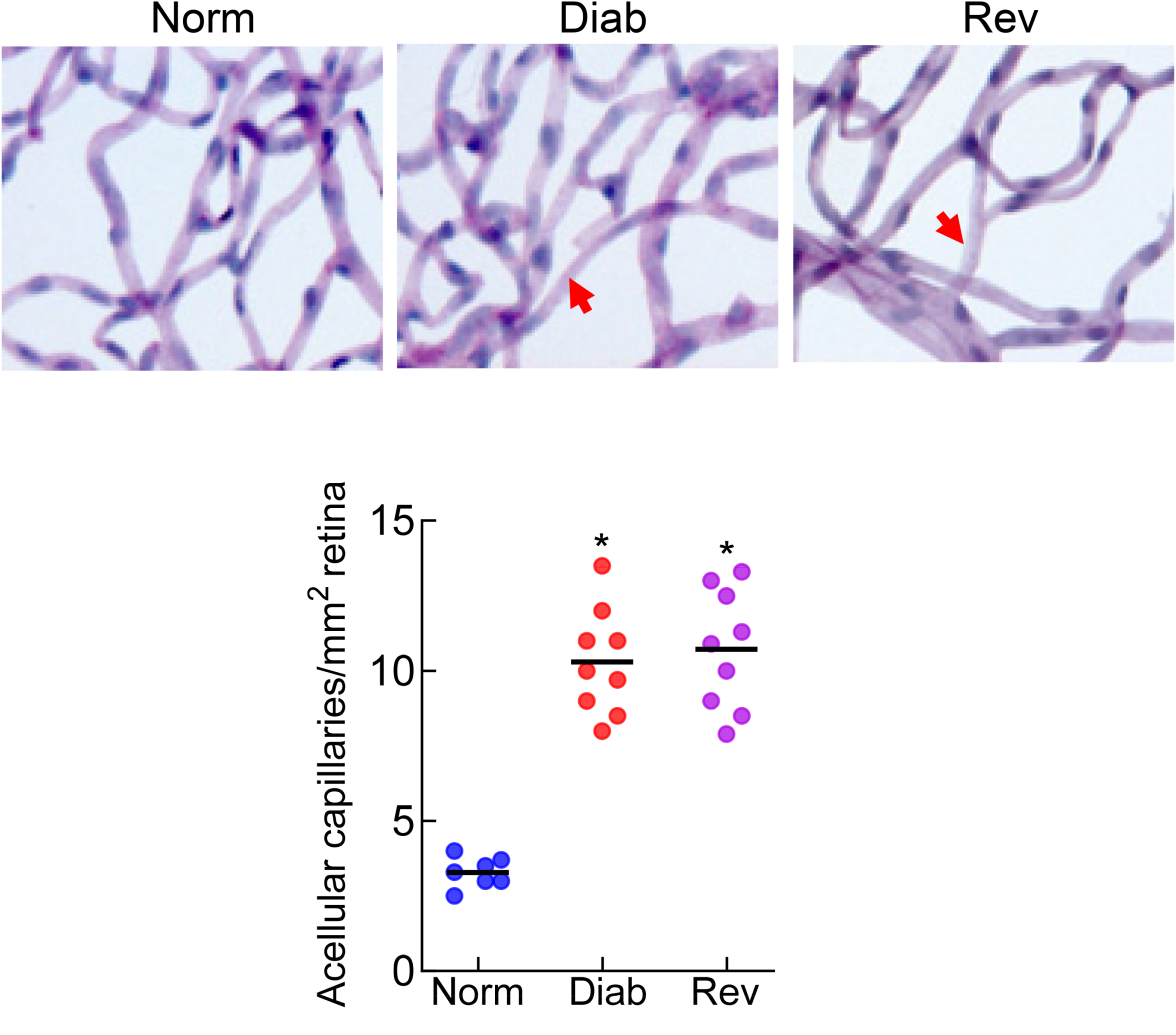
Reinstitution of good glycemic control in diabetic rats and retinal histopathology. Acellular (degenerative) capillaries were counted in the entire periodic acid‐Schiff–hematoxylin‐stained trypsin‐digested retinal microvasculature. The arrow indicates an acellular capillary. The values in the accompanying graph are mean ± SD from 7 to 9 rats in each group. Diab, rats in continuous poor glycemic control for 8 months; Norm, normal control rats; Rev, rats in poor glycemic control for 4 months, followed by good glycemic control for four additional months. *p < 0.05 versus Norm group.

Compared with normal rats (Norm group), body weight of rats maintained in continuous poor control for 8 months (Diab group) was >30% lower, and glycated hemoglobin and urine output were twofold and approximately ninefold, respectively, higher (*p* < 0.05). Rats in the Rev group, during their poor control period had similar body weight, glycated hemoglobin, and urine output values as the rats in the Diab group (*p* > 0.05). However, four additional months of good glycemia in these rats resulted in the values that were not different from the normal rats (*p* > 0.05; Table 2). The number of degenerative capillaries in the retinal vasculature, a hallmark of diabetic retinopathy, in the Diab group was approximately threefold higher, compared with the Norm group, and in the Rev group, the values were similar to those in the Diab group (Figure 1, *p* > 0.05 vs. Diab).

**TABLE 2:** Severity of hyperglycemia in rats.

Group	Body weight (g)	Blood glucose (mg/dL)	GHb (%)	Urine Volume (mL/24 h)
Norm	575 ± 41	105 ± 26	5.6 ± 0.98	15 ± 6
Diab	380 ± 59*	475 ± 70*	10.3 ± 1.5*	125 ± 49*
Rev (PC → GC)	401 ± 34* → 539 ± 43	459 ± 61* → 139 ± 36	9.6 ± 1.1* → 5.9 ± 1.0	110 ± 37* → 25 ± 10

*Note*: Body weight and blood glucose were measured once every week, 24‐h urine output once every other week and glycated hemoglobin (GHb) every other month. Values obtained during the entire study are represented as mean ± SD from 6 to 7 rats/group.

Abbreviations: Diab, rats in continuous poor glycemic control for 8 months; Norm, normal control; Rev, rats in poor glycemic control (PC) for 4 months, followed by good glycemic control (GC) for four additional months.

* *p* < 0.05 versus Norm.

### 3.2 Differentially expressed LncRNAs

**FIGURE 2:**
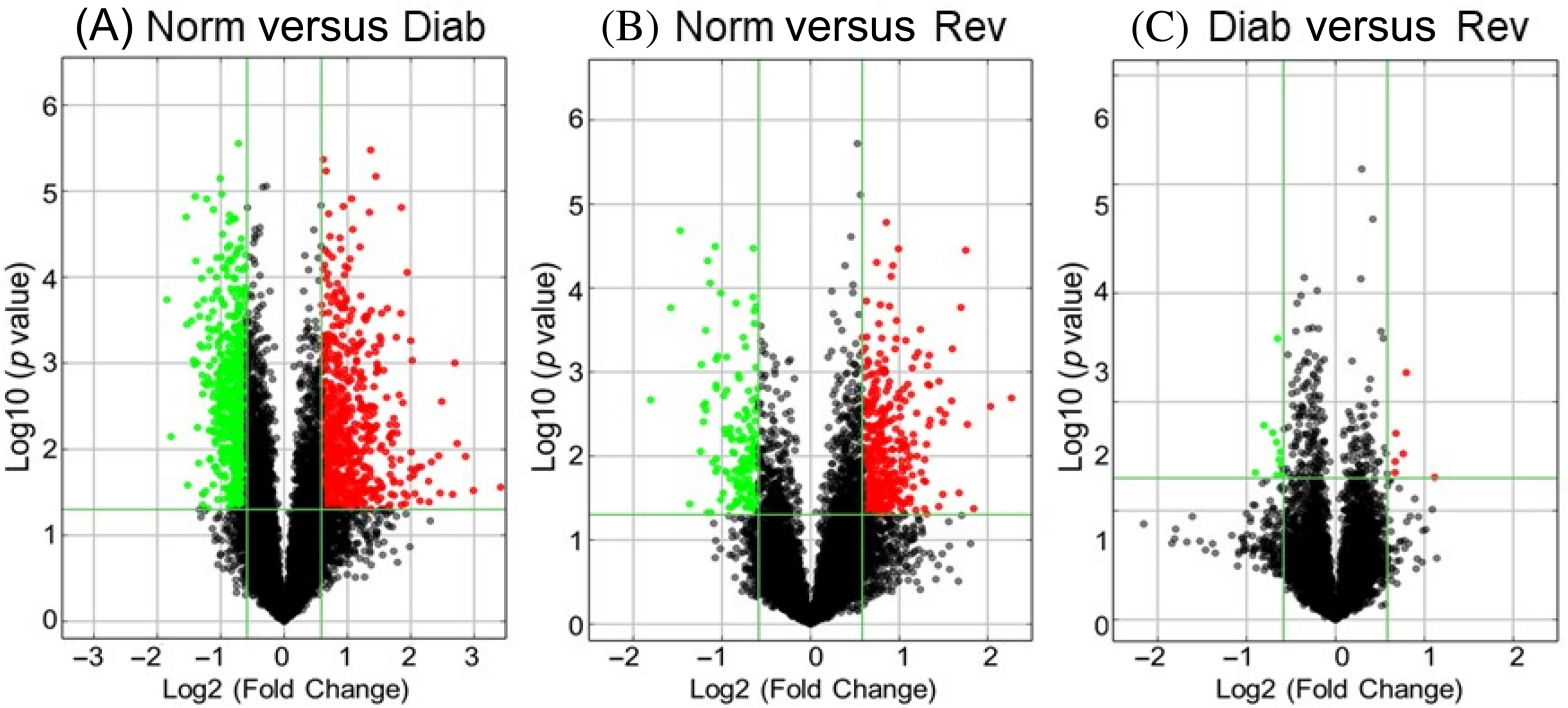
Retinal long noncoding RNAs (LncRNAs) microarray profiling and reversal of hyperglycemia. Representative Volcano plots illustrating differentially expressed LncRNAs between (A) normal control and in continuous poor glycemic control for 8 months (Diab group), (B) Norm group and in poor glycemic control for 4 months, followed by good glycemic control for four additional months (Rev group), and (C) Diab and Rev groups. Vertical lines correspond to more than twofold downregulation or upregulation, and the horizontal lines represent a value of *p* < 0.05.

**FIGURE 3:**
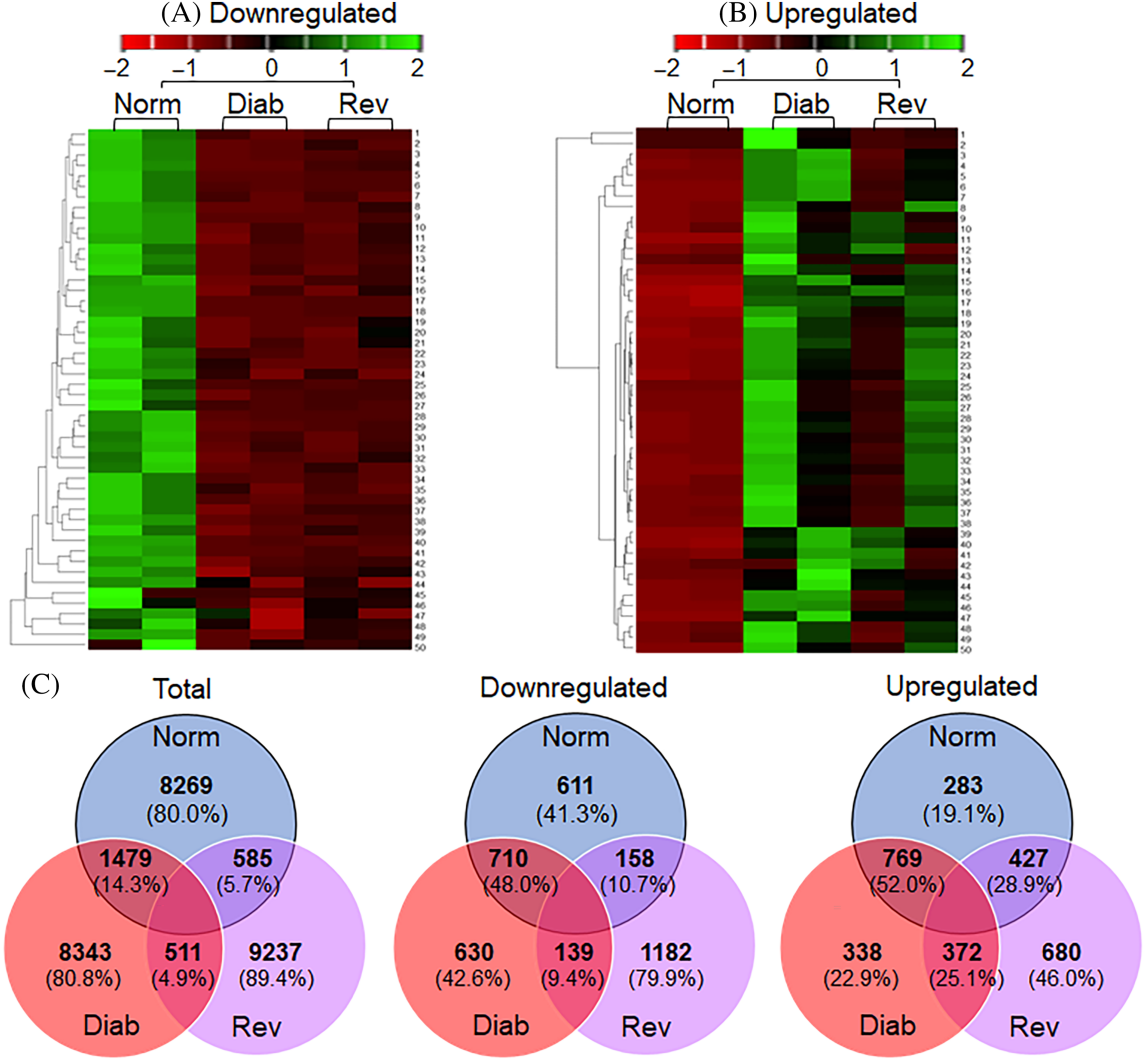
Heat maps representing expression profiles of differentially expressed long noncoding RNAs (LncRNAs). Representative heat maps from two rats in each group, generated from the hierarchical cluster analysis showing the expression profiling of top 50 retinal (A) downregulated LncRNAs (1‐Csnk1d, 2‐Ankef1, 3‐LOC102546306, 4‐ENSRNOG00000059416, 5‐ENSRNOG00000053967, 6‐ENSRNOG00000055794, 7‐ENSRNOG00000061604, 8‐LOC102550778, 9‐LOC102549422, 10‐LOC103691014, 11‐LOC102555927, 12‐LOC102546437, 13‐ENSRNOG00000004900, 14‐LOC102553204, 15‐Slfn4, 16‐LOC102548149, 17‐ENSRNOG00000051329, 18‐LOC103691740, 19‐ENSRNOG00000055811, 20‐LOC102550778, 21‐LOC102546999, 22‐Ankle1, 23‐Gapdh‐ps1, 24‐LOC103694005, 25‐ENSRNOG00000053156, 26‐LOC102554852, 27‐LOC103692120, 28‐ENSRNOG00000053062, 29‐ENSRNOG00000018191, 30‐LOC102554566, 31‐LOC103691745, 32‐LOC102556677, 33‐LOC102551010, 34‐LOC102548573, 35‐ENSRNOG00000057973, 36‐LOC102553583, 37‐ENSRNOG00000059467, 38‐LOC102546336, 39‐LOC103694189, 40‐LOC102552701, 41‐LOC102550026, 42‐ENSRNOG00000058323, 43‐LOC102546992, 44‐LOC102550324, 45‐LOC102549385, 46‐LOC102546527, 47‐LOC102555980, 48‐LOC103692978, 49‐LOC100362486, and 50‐LOC103693482) and (B) upregulated LncRNAs (1‐LOC103691123, 2‐LOC102553726, 3‐Efcab1, 4‐LOC100912209, 5‐LOC100912209, 6‐ENSRNOG00000060152, 7‐Tchhl1, 8‐ENSRNOG00000050090, 9‐uc.298, 10‐LOC102547518, 11‐LOC102552912, 12‐ENSRNOG00000010676, 13‐Prpf4b, 14‐ENSRNOG00000061342, 15‐ENSRNOG00000061365, 16‐LOC102555442, 17‐ENSRNOG00000053302, 18‐ENSRNOG00000056575, 19‐ENSRNOG00000062118, 20‐LOC102553769, 21‐LOC102547871, 22‐LOC102553769, 23‐LOC103694026, 24‐LOC102553522, 25‐ENSRNOG00000054077, 26‐ENSRNOG00000055686, 27‐LOC102548125, 28‐LOC102553486, 29‐ENSRNOG00000051834, 30‐LOC102553612, 31‐Slc7a6, 32‐ENSRNOG00000017045, 33‐ENSRNOG00000060101, 34‐LOC103693073, 35‐LOC102555506, 36‐LOC102549835, 37‐LOC102557424, 38‐ENSRNOG00000058192, 39‐LOC102550786, 40‐ENSRNOG00000028856, 41‐uc.224, 42‐uc.247, 43‐LOC103692927, 44‐ENSRNOG00000059384, 45‐ENSRNOG00000053021, 46‐uc.243, 47‐LOC103691428, 48‐LOC102554555, 49‐LOC102548736, and 50‐LOC103691846) in Norm, Diab and Rev groups. The color scale on the top illustrates the *Z*‐score to represent the relative expression of LncRNAs; red indicating relatively “low” and green relatively “high” expression, compared with Norm group. (C) Venn diagrams demonstrating mutually expressed total, downregulated and upregulated retinal LncRNAs in Norm, Diab, and Rev groups. Diab, continuous poor glycemic control for 8 months; Norm, normal control; Rev, poor glycemic control for 4 months, followed by good glycemic control for four additional months.

A total of 10 333 LncRNAs were identified by Arraystar, and based on >2‐fold change, a *p* value less than 0.05 and Hierarchical clustering, 1479 differentially expressed LncRNAs were identified between the Norm and Diab groups. Among the differentially expressed LncRNA, although, 710 were downregulated and 769 were upregulated in Diab groups. Between the Norm and Rev groups, 585 LncRNAs were differentially expressed, and among those, 158 were downregulated and 427 were upregulated. Between the Diab and Rev groups, 139 common LncRNAs were downregulated and 372 were upregulated, suggesting their importance in the metabolic memory phenomenon. Figure 2A–C shows the volcano plots of differentially expressed LncRNAs between the Norm and Diab, Norm and Rev, and Diab and Rev groups, respectively. Heat maps of the downregulated, and of the upregulated, LncRNAs in the three experimental groups are presented in Figure 3A,B. Venn diagram showing mutually expressed downregulated and upregulated LncRNAs between the Norm and Diab, Norm and Rev, and Diab and Rev groups are presented in Figure 3C. The left panel depicts all of the LncRNAs identified in the three groups, and while the middle panel shows only the number of LncRNAs that were significantly downregulated compared to Norm group, the right panel shows upregulated LncRNAs.

**FIGURE 4:**
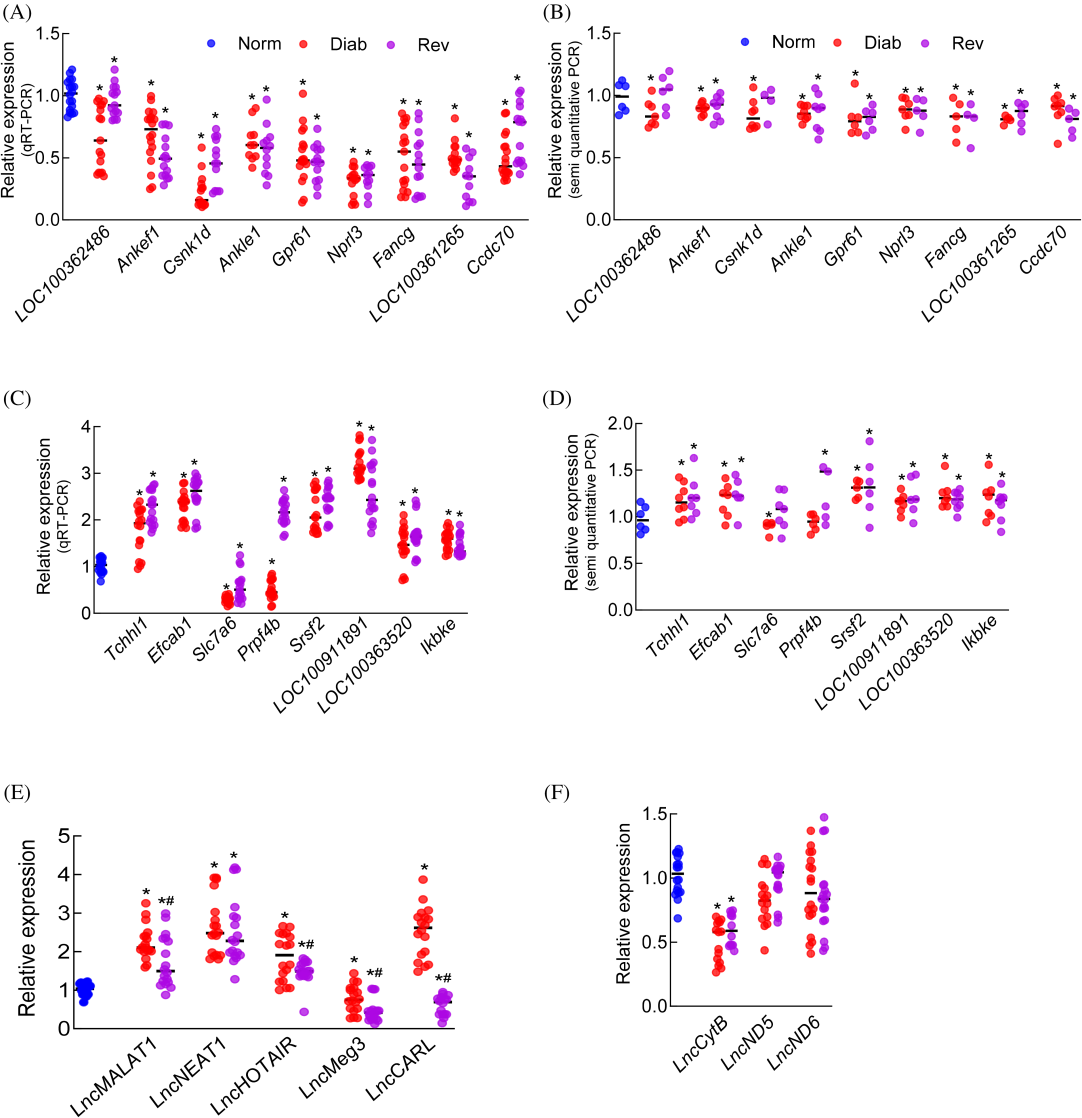
Validation of relative expression of differentially expressed long noncoding RNAs (LncRNAs). (A and B) Downregulated and (C and D) upregulated LncRNAs, validated by quantitative real‐time polymerase chain reaction (qRT‐PCR) and semiquantitative PCR. Expression of the known LncRNAs implicated in diabetic retinopathy, encoded by (E) nuclear genome and (F) mitochondrial genome. Values are mean ± SD from 6 to 7 rats/group, with each measurement made in triplicate. Blue denotes normal control rats; red, rats in continuous poor glycemic control; and purple, rats in poor glycemic control for 4 months, followed by good glycemic control for four additional months. *p < 0.05 versus Norm group.

A group of top differentially expressed LncRNAs that have been shown to play a role in various metabolic functions^31^ were further validated by qRT‐PCR and semiquantitative PCR (Table S1 presents details about these LncRNAs). The nine LncRNAs, downregulated in both in the Diab and Rev groups, compared with the Norm group, identified by microarray analysis, were confirmed as downregulated by both qRT‐PCR and semiquantitative PCR analyses (Figure 4A,B). In the Rev group, other than Lnc*LOC100362486*, which remained unchanged, rest of the same eight LncRNAs were downregulated, as confirmed by the PCR analyses. Among the eight upregulated LncRNAs identified by Array analysis, PCR analyses (quantitative and semiquantitative) showed downregulation (or no change) of Lnc*Slc7a6* in both Diab and Rev groups, and while upregulation of Lnc*Prpf4b* in Rev group, its downregulation in Diab group (Figure 4C,D).

To further validate, LncRNAs implicated in the pathogenesis of diabetic retinopathy^20^, ^21^ were also quantified by qRT‐PCR. Compared with Norm, in Diab and Rev groups, LncRNAs *MALAT1*, *NEAT1*, and *HOTAIR* were upregulated and Lnc*Meg3* was downregulated. However, while Lnc*CARL* was significantly upregulated in Diab group, it was downregulated in Rev group (Figure 4E).

Although LncRNAs are mainly encoded by nuclear genome, mitochondrial DNA (mtDNA) also encodes three major LncRNAs, Lnc*CytB*, Lnc*ND5* and Lnc*ND6*.^32^ The effect of reversal of hyperglycemia on mtDNA‐encoded LncRNAs was also quantified using strand‐specific PCR. As expected,^23^ compared with the Norm group, Lnc*CytB* expression was downregulated in the Diab group (*p* < 0.05), and Lnc*ND5* and Lnc*ND6* were unchanged. Consistent with the Diab group, Lnc*CytB* was the only mtDNA‐encoded LncRNA downregulated in the Rev group (Figure 4F).

### 3.3 Regulatory role(s) of LncRNAs by pathway and GO analysis

LncRNAs regulate genes that are in their close proximity and also interact with chromatin at many different locations modifying the gene expression.^14^, ^15^ To understand the functional role of LncRNAs in the metabolic memory phenomenon, GO and pathway analyses of LncRNAs and mRNAs were performed. Since overall characterization of LncRNAs is poor, using GO resources.

**FIGURE 5:**
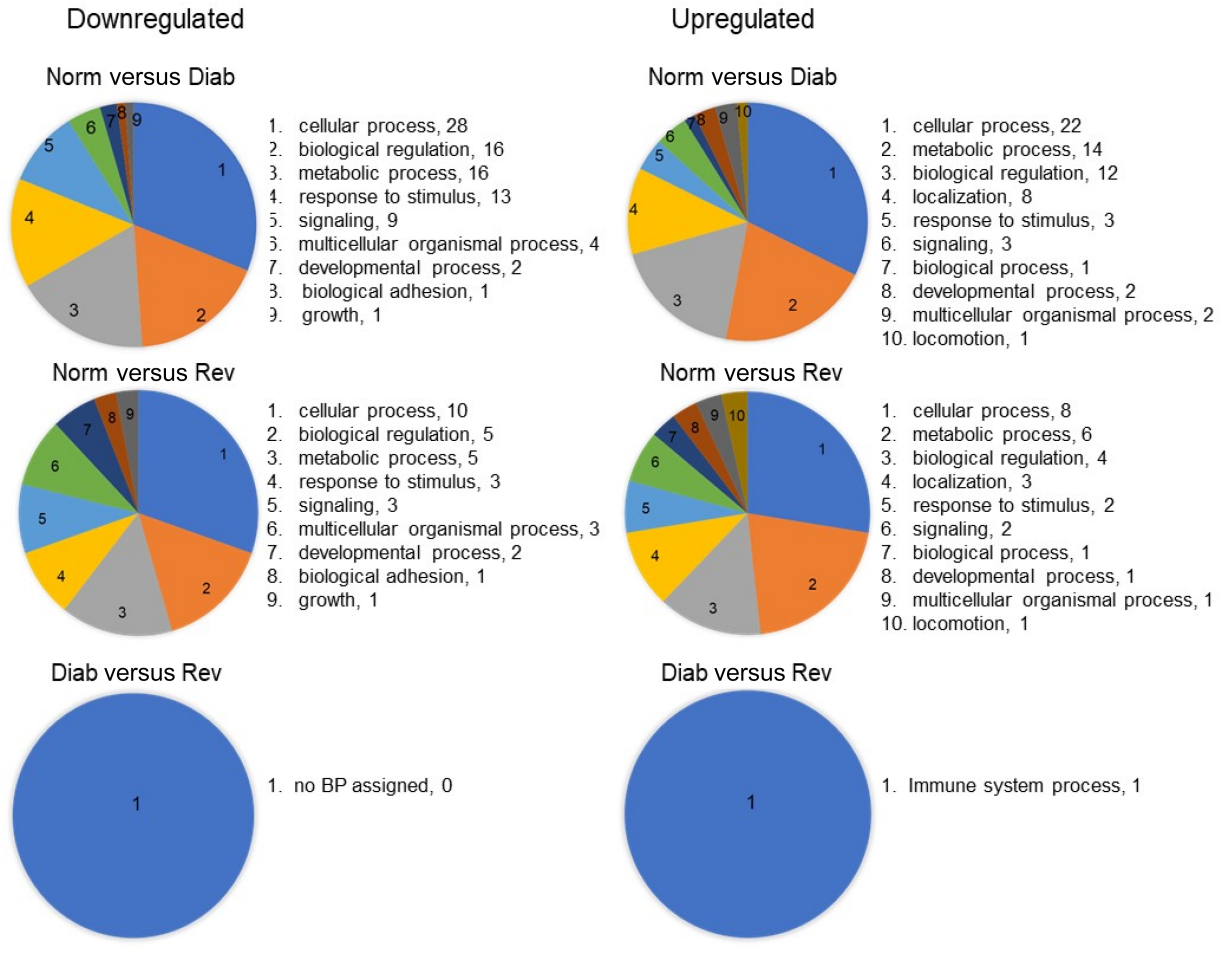
Pie charts showing gene ontology biological process (BP) associated with the function of the downregulated and the upregulated LncRNAs between the Norm versus Diab, Norm versus Rev, and Diab versus Rev groups. Diab, rats in continuous poor glycemic control for 8 months; Norm, normal control rats; Rev, rats in poor glycemic control for 4 months, followed by good glycemic control for four additional months.

GO analysis demonstrated involvement of few LncRNA in a limited number of biological processes; in the Diab group, only 12 biological processes could be associated with the 710 downregulated LncRNAs and 27 with the 769 upregulated LncRNAs. In the Rev group, 13 biological processes had some association with the 158 downregulated LncRNAs and nine with the 427 upregulated LncRNAs. However, between the Diab and Rev groups, we could not identify any biological process for the 10 downregulated LncRNAs, and only one, associated with immune system processes, was identified for the six upregulated LncRNAs (Figure 5).

**FIGURE 6:**
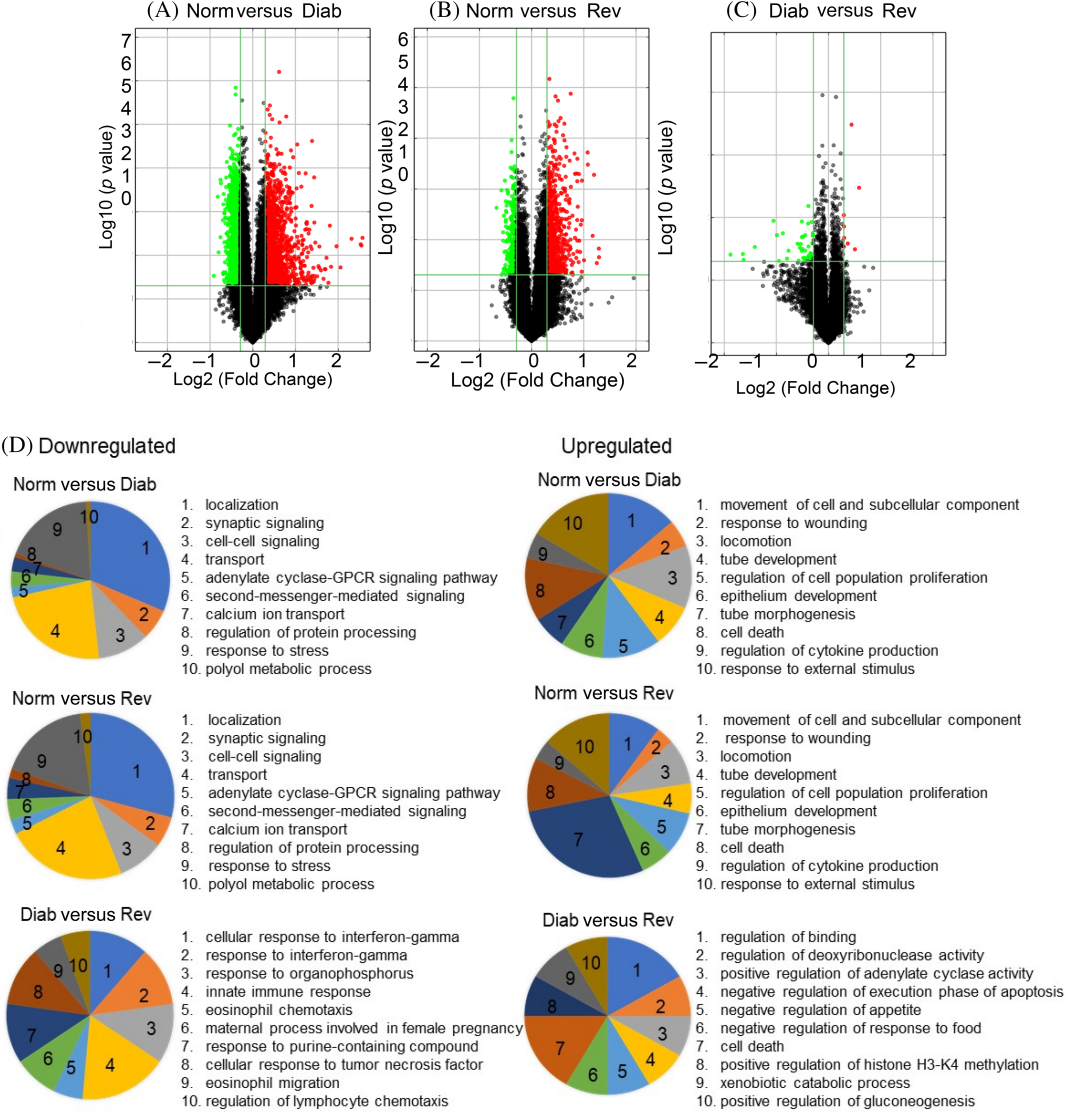
Profiling of mRNAs and reversal of hyperglycemia. (A–C) show representative Volcano plots between the Norm and Diab, Norm and Rev, and Diab and Rev groups, respectively. (D) Gene ontology biological process classification of differentially expressed mRNAs. Pie charts for the top 10 gene ontology terms associated with gene function of the downregulated and the upregulated mRNAs in the Norm versus Diab, Norm versus Rev, and Diab versus Rev groups. Top biological processes were selected according to the enrichment score in the decreasing order.

Compared with 10, 333 LncRNAs, the Arraystar method identified 27, 346 mRNAs, and among those, 3566 mRNAs were differentially expressed between the Norm and Diab groups (1806 downregulated and 1760 were upregulated) and 1088 between the Norm and Rev groups (349 downregulated and 739 were upregulated). Further analysis identified 1060 common mRNAs that were differentially expressed in both the Diab and Rev groups, and among those, 303 were downregulated and 657 were upregulated (Figure 6A–C). GO analysis based on biological processes using all of the differentially expressed mRNA identified 2412 biological processes with differential regulation in the Diab group, compared with the Norm group, and among those, 1047 were downregulated and 1365 were upregulated. Between the Norm and Rev groups, 668 biological processes showed differential expression with 361 downregulated and 307 upregulated. Interestingly, 368 biological processes were common in the Diab and Rev groups including 165 downregulated and 203 upregulated. These biological processes are mainly associated, and implicated, in cell–cell signaling, cell proliferation, tube development, stress response, mitochondrial damage, cell death, and cytokine production. However, 514 biological processes were different between the Diab and Rev groups including 372 downregulated and 142 upregulated (Figure 6D).

**FIGURE 7:**
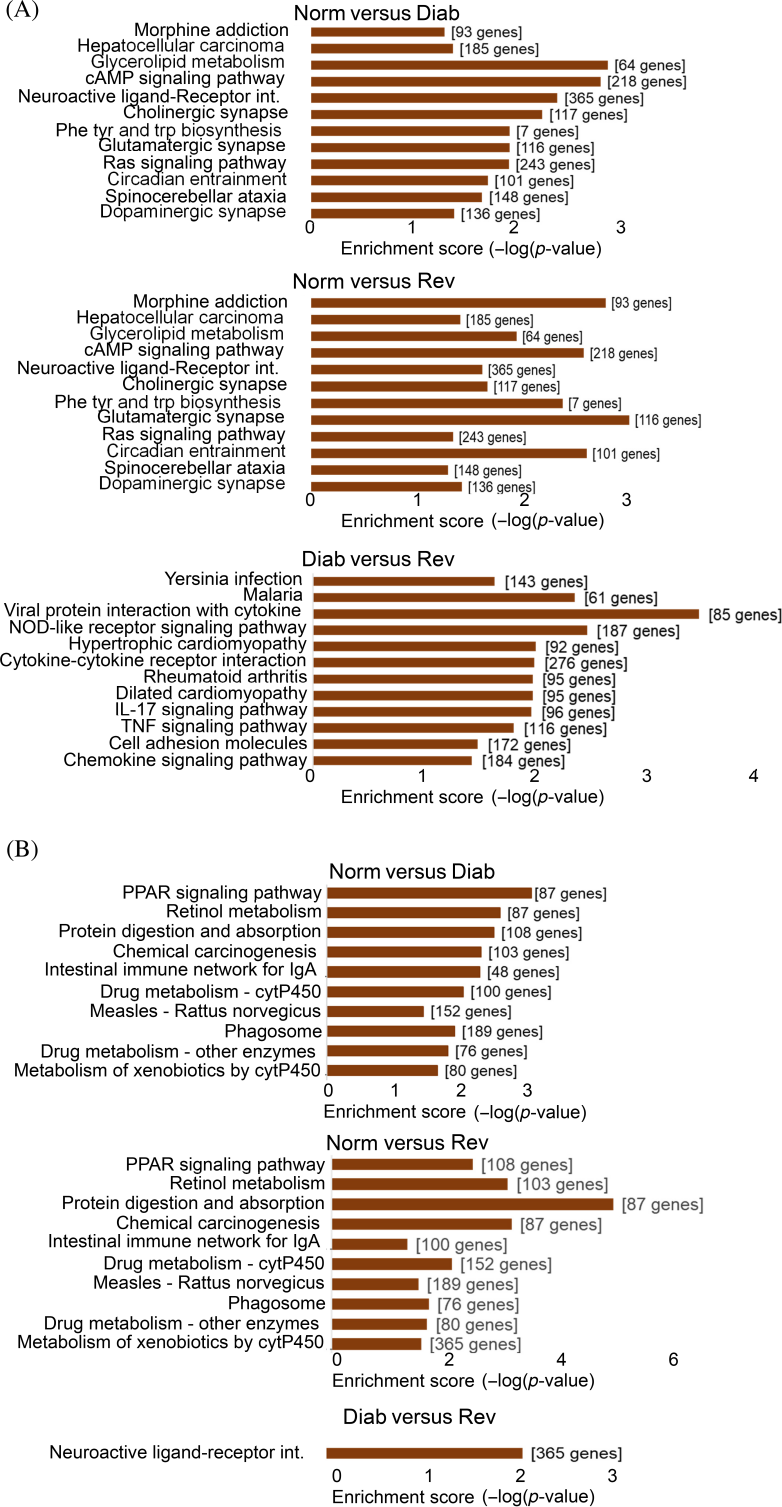
Kyoto Encyclopedia of Genes and Genomes (KEGG) pathways analysis of differentially expressed genes. KEGG pathways corresponding to the retinal mRNAs (A) downregulated and (B) upregulated, in rats maintained in continuous poor glycemic control for 8 months (Diab group), rats maintained in good glycemic control for 4 months after 4 months of poor glycemic control (Rev group), compared with normal control rats (Norm group), and the Diab and Rev groups. Top KEGG pathways were selected according to the enrichment score in the decreasing order.

Although KEGG pathway analysis demonstrated involvement of a few LncRNAs in the biological pathways (Figure S2), analysis of differentially expressed mRNA, however, identified 90 pathways between the Norm and Diab groups (52 downregulated and 38 upregulated), and 38 pathways between the Norm and Rev groups (22 downregulated and 16 upregulated). Among those pathways, 22 common pathways were determined between the Diab and Rev groups, which included 12 downregulated and 10 upregulated. Interestingly, 14 pathways (13 downregulated and 1 upregulated), that were not identified as common in the Norm and Diab or the Norm and Rev groups, were also identified as common pathways between the Diab and Rev groups (Figure 7A,B). These pathways are mainly associated with Ras signaling, cyclic adenosine monophosphate (cAMP) signaling, retinol metabolism, apoptosis, glycerolipid metabolism, protein digestion, and coagulation cascades.^33^

### 3.4 LncRNA–mRNA coexpression network

Interactions among the differentially expressed 51 LncRNAs (*p* < 0.01 vs. the Norm group), and 50 differentially expressed mRNAs (implicated in various metabolic abnormalities implicated in diabetic retinopathy including oxidative stress, mitochondrial function and cell division), a coexpression network was constructed. Figure S2 shows the entire coexpression network profile consisting of 101 network nodes, 731 connections among 50 differentially expressed mRNAs (orange node) and 51 differentially expressed LncRNAs (blue node). Among the selected mRNA and LncRNAs, 731 interactions, including 393 positive (solid line) and 338 negative correlations (equal dash line), were identified, and analysis between the Norm and Diab groups and the Norm and Rev groups demonstrated coexpression of the similar selected mRNA with LncRNAs (Figure S2).

**FIGURE 8:**
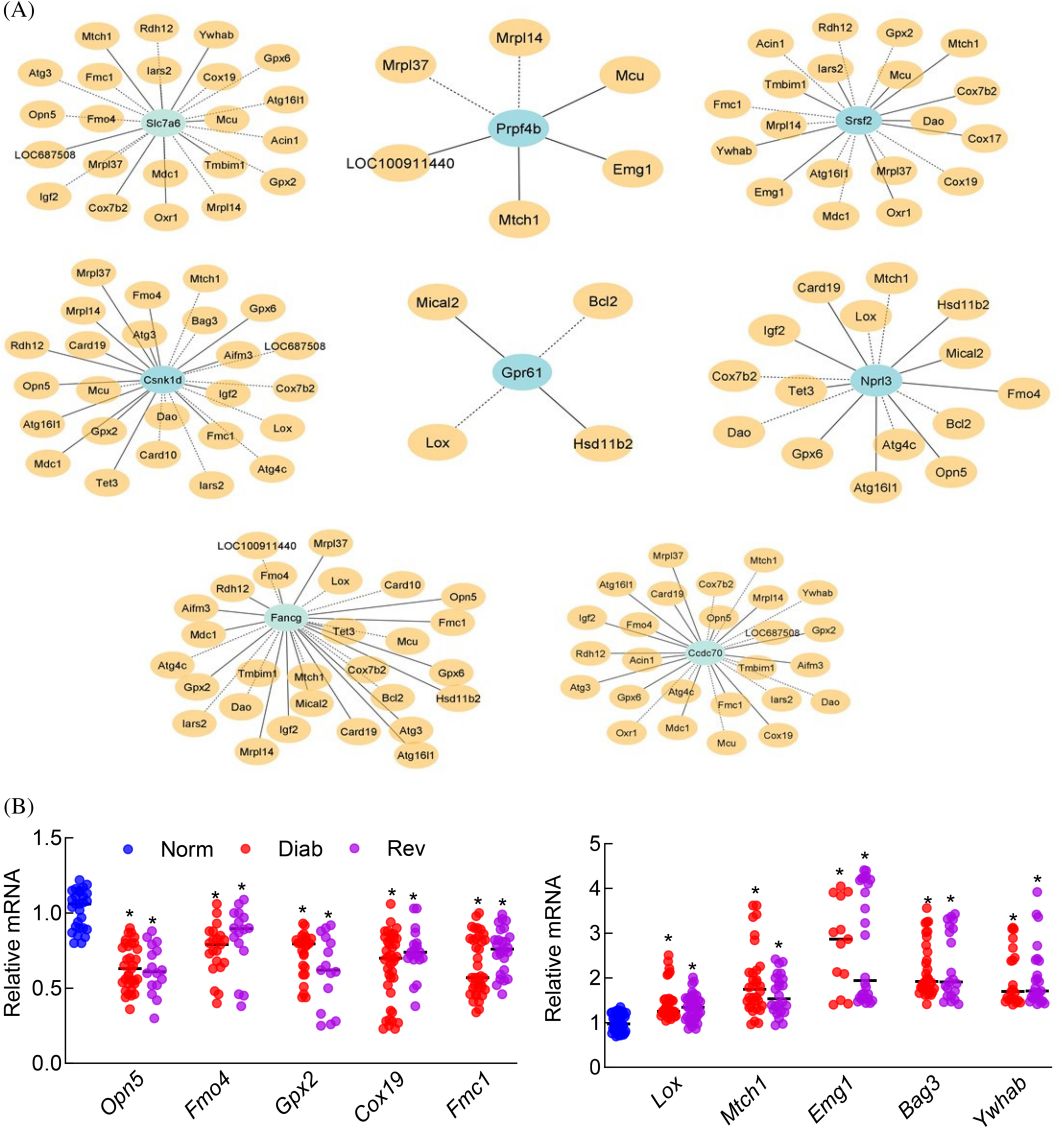
Long noncoding RNAs (LncRNA)–mRNA coexpression network of selected LncRNA with mRNAs. (A) Coexpression network of differentially expressed LncRNAs. Blue node denotes LncRNA; orange node denotes mRNA coding gene; solid line denotes directed, positive correlation; broken line, undirected, negative correlation. (B) Gene transcripts, quantified by quantitative real‐time polymerase chain reaction (qRT‐PCR) using β‐actin as a housekeeping gene for downregulated and upregulated mRNAs. Values are represented as mean ± SD from 6 to 7 rats/group. Diab, continuous poor glycemic control for 8 months; Norm, normal control; Rev, poor glycemic control for 4 months, followed by good glycemic control for 4 months. *p < 0.05 versus Norm.

LncRNA–mRNA coexpression was further confirmed in selected eight common LncRNAs from the list showing significantly different expression in both the Diab and Rev groups (*p* < 0.05 vs Norm). As identified by the microarray analysis and LncRNA‐mRNA network, LncRNA *Slc7a6* was correlated with 22 mRNAs, *Prpf4b* with five, *Srsf2* with 19, *Csnk1d* with 25, *Gpr61* with four, *Nprl3* with 15, *Fancg* with 28, and *Ccdc70* with 26 mRNAs (Figure 8A). qRT‐PCR analysis of 10 of the randomly selected mRNAs showing coexpression with these LncRNAs produced similar downregulation or upregulation pattern in the Diab and Rev groups (Figure 8B).

## 4 DISCUSSION

Proteins are coded by about only 2% of the human genome, and noncoding RNAs are approximately four times more than the coding RNA sequences.^13^, ^15^, ^17^ Noncoding RNAs are not translated into proteins, but they can regulate gene expression at the transcriptional and posttranscriptional levels. Among the major noncoding RNAs, >30 000 LncRNAs have been discovered in humans thus far with their possible role in remodeling chromatin, genomic imprinting and regulating gene expression, but the LncRNAs field is still in its early stages.^34^ Recent technological advancements have documented aberrant expression of several LncRNAs in many diseases including cancer and diabetes.^18^, ^20^, ^21^ A number of retinal LncRNAs including *MALAT1*, *MIAT1*, *ANRIL*, and *CytB* are also aberrantly expressed in diabetes, and are associated with the regulation of metabolic abnormalities implicated in the development of diabetic retinopathy including inflammation, oxidative stress and mitochondrial damage.^20^, ^21^, ^22^, ^23^ Continued progression of diabetic retinopathy, even after maintenance of tight glycemic control, is a problem faced by many diabetic patients^35^, ^36^; using a rat model of metabolic memory,^5^, ^6^, ^8^, ^12^, ^37^ we present the first report showing that several retinal LncRNAs also continue to be aberrantly expressed after termination of hyperglycemia, suggesting their role in the metabolic memory phenomenon.

Profiling of LncRNAs in streptozotocin‐induced mice, diabetic for 2 months, have identified more than 300 aberrantly expressed retinal LncRNAs including 214 downregulated and 89 upregulated.^38^ Furthermore, the fibrovascular membranes of patients with diabetic retinopathy have shown over 420 differentially expressed LncRNAs, 263 upregulated and 164 downregulated.^39^ Here, we present 1479 retinal LncRNAs with aberrant expression between the Norm and Diab groups (710 downregulated and 769 upregulated), and among those, 511 common LncRNAs in the Diab and Rev groups (139 downregulated and 372 upregulated), suggesting the importance of these common LncRNAs in the metabolic memory phenomenon. For example, overlapping gene associated with downregulated exon‐sense intergenic LncRNA *Ankle1*, associated with DNA endonuclease activity and DNA recombination, Lnc*Fancg* with DNA damage response,^40^ and Lnc*Srsf2* with the overlapping gene associated with protein kinase C activity,^31^ remain aberrantly expressed after hyperglycemia termination. In accordance, retinal mtDNA biogenesis/damage/repair and protein kinase C activity remain compromised even after termination of hyperglycemia.^41^, ^42^, ^43^ Our data also show that upregulated LncRNAs *MALAT1*, *NEAT1*, and *HOTAIR* and downregulated Lnc*Meg3* in diabetes do not benefit from hyperglycemia termination; these LncRNAs are implicated in the regulation of various abnormalities associated with the development of diabetic retinopathy including cellular antioxidant defense system, mitochondria function, inflammation, vascular permeability, angiogenesis, and cell proliferation.^18^, ^19^, ^20^, ^21^, ^22^, ^23^ Importance of continued aberrant expression of these LncRNAs is supported by previous studies showing the failure of oxidative stress, inflammation, and mitochondrial damage to benefit from reversal of hyperglycemia.^5^, ^6^, ^8^, ^12^

Mitochondrial dysfunction plays a central role in the development of diabetic complications.^11^, ^44^ In diabetic retinopathy, retinal mitochondrial functional, structural and genomic stability is impaired, culminating in accelerated capillary cell apoptosis, a phenomenon which precedes the development of histopathology characteristic of diabetic retinopathy.^11^ Mitochondrial genome encodes three major LncRNAs, and these antisense LncRNAs are located complementary to their gene encoding regions for *ND5*, *ND6 of complex I and cytochrome B of complex III*, respectively.^32^ In diabetic retinopathy, although the expression of Lnc*ND5* and Lnc*ND6* is not altered, Lnc*CytB* is downregulated, impacting mitochondrial functional and genomic stability.^23^ Here, our results show that Lnc*CytB* remains downregulated after hyperglycemia termination, implying its role in the metabolic memory phenomenon. In‐line with continued Lnc*CytB* downregulation, mitochondrial function, structure, and genome remain compromised when hyperglycemia is reversed by normal glycemia, and histopathological lesions of diabetic retinopathy fail to reverse.^10^, ^11^

Although LncRNAs do not code for proteins, recent technological advancements have demonstrated their role in the regulation of gene expression, especially by post‐transcriptional and epigenetic mechanisms, including chromatin remodeling, mRNA splicing modulation and decay and genomic imprinting.^14^, ^15^, ^17^ They can also act as scaffolds of protein complexes, sponges for miRNAs or regulators of epigenetic factors.^45^ Our GO and pathway analysis of LncRNAs and mRNA show a very few LncRNAs involved in biological processes; this could be due to poor, or no, characterization of most of the LncRNAs identified by the microarray analysis and lack of existing database for their functional annotations. But, the systematic analysis of the functions of differentially expressed mRNAs by GO annotation and pathway analysis, based on biological processes/molecular function/ cellular component identified over 160 same downregulated mRNAs and over 200 upregulated mRNA. These mRNAs are mainly associated with biological process implicated in cell–cell signaling, cell proliferation, tube development, stress response, cell death, and cytokine production.^46^ The importance of these mRNAs in metabolic memory is consistent with previous studies showing that increase in retinal capillary apoptosis and inflammatory mediators, initiated by hyperglycemia, do not benefit from the normoglycemia which follows.^10^, ^47^ Furthermore, the coexpression network analysis demonstrates coexpression of similar selected LncRNAs with mRNAs between normal and diabetic rats, either in continuous poor glycemia (Diab group) or after reversal of their poor glycemia (Rev group). These mRNAs are involved in oxidative stress, mitochondrial dysfunction, cell division, transport, autophagy, and apoptosis.^46^, ^48^ In support, these pathways are implicated in the complex and multifactorial etiology of diabetic retinopathy, and also in the metabolic memory associated with its continued progression.^10^, ^12^, ^37^, ^42^, ^43^, ^47^

We acknowledge that our analysis is based on a small number of LncRNAs, this is because the LncRNA field is still in the early stages and most of the LncRNAs detected by the microarray are not yet characterized.^49^ The microarray analyses failed to identify some nuclear DNA and mtDNA‐encoded LncRNAs that have been implicated in diabetic retinopathy including *NEAT1*, *HOTAIR*, and *CytB*. This could be due to the limitation of the technique, as microarray technique profiles predefined transcripts through hybridization and quantifies the expression levels of each transcript by reading out intensities of hybridization signals, and does not allow for the sequencing of the whole transcriptome. Furthermore, we cannot rule out the possibility that due to the limitation of the Microarray technique in not covering all isoforms of LncRNAs, we could have missed some important LncRNAs. Validation of our microarray data by PCR has presented discrepancy in Lnc*Slc7a6* and Lnc*Prpf4b* expression among the groups, this could be due to the differences in the sensitivity of these two methods; compared to PCR, microarray technique is more sensitive because PCR technique can be influenced by amplification biases, exponential amplification of errors or change in efficiency of qPCR at later cycles. In addition, the differences in data normalization, for example, microarray analysis by global normalization and PCR by using candidate reference gene, contributing to these differences,^50^ cannot be ruled out. However, consistent results from both quantitative and semiquantitative PCR techniques further strengthen our findings.

In conclusion, metabolic memory associated with the continued progression of diabetic retinopathy remains a nagging problem for many diabetic patients striving to maintain good glycemic control. Although LncRNA field is not as advanced as miRNA field, recent technological advancements have uncovered many regulatory LncRNAs important in biological processes in both health and disease. Using animal model of metabolic memory in which diabetic retinopathy does not benefit from hyperglycemia termination, this study has identified retinal LncRNAs that continue to express aberrantly even when hyperglycemia is terminated. Our microarray profiling data for LncRNAs not only provide a better understanding of their underlying molecular mechanisms, but has also identified new LncRNAs and their gene targets that could be playing a crucial role in the continued progression of diabetic retinopathy including oxidative stress, inflammation and mitochondrial damage. Focusing on these LncRNAs will provide a strong background for their use as potential therapeutic targets to slow down the development/ progression of this sight‐threatening disease.

## AUTHOR CONTRIBUTIONS

JK planned the experiment, researched and interpreted the data, and contributed to manuscript editing; PM researched data and contributed to manuscript editing; RAK contributed to experimental plan, data interpretation, literature search, manuscript writing and editing.

## FUNDING INFORMATION

This work was supported in parts by grants from the National Institutes of Health (EY017313 and EY333516) and from The Thomas Foundation to RAK, and an unrestricted grant from Research to Prevent Blindness to the Ophthalmology Department.

## CONFLICT OF INTEREST STATEMENT

The authors declare no conflicts of interest.

## Supporting information


**Figure S1.** KEGG pathways analysis of differentially expressed LncRNAs. Top KEGG pathways corresponding to (A) downregulated and (B) upregulated retinal LncRNAs in Diab versus Norm and Rev versus Norm groups.
**Figure S2.** LncRNA–mRNA coexpression network. Coexpression network of 51 differentially expressed retinal LncRNAs and 50 differentially expressed mRNAs, constructed using LncRNA–mRNA pairs with Pearson's correlation coefficient >0.9 and *p* < 0. 01, in rats maintained in continuous poor glycemic control for 8 months (Diab group). Blue nodes = LncRNAs; Orange nodes = mRNA coding genes; Solid line = Directed, positive correlation and broken line = Undirected, negative correlation.

**Table S1.** Functional details of LncRNAs.

## Data Availability

RAK is the guarantor of this work and takes responsibility for the integrity of the data and the accuracy of the data analyses. Raw and processed Microarray datasets generated during the current study are submitted in the Gene Expression Omnibus, GSE251731, and are also available from the corresponding author.
