# Supplementary material for: Long noncoding RNAs and metabolic memory associated with continued progression of diabetic retinopathy
Source: J Diabetes. 2024 Nov 18;16(11):e70009. doi: 10.1111/1753-0407.70009 (PMC11574110; doi:10.1111/1753-0407.70009)

Figure S1. KEGG pathways analysis of differentially expressed LncRNAs.

Top KEGG pathways corresponding to (A) downregulated and (B) upregulated retinal LncRNAs in Diab vs Norm and Rev vs Norm groups.

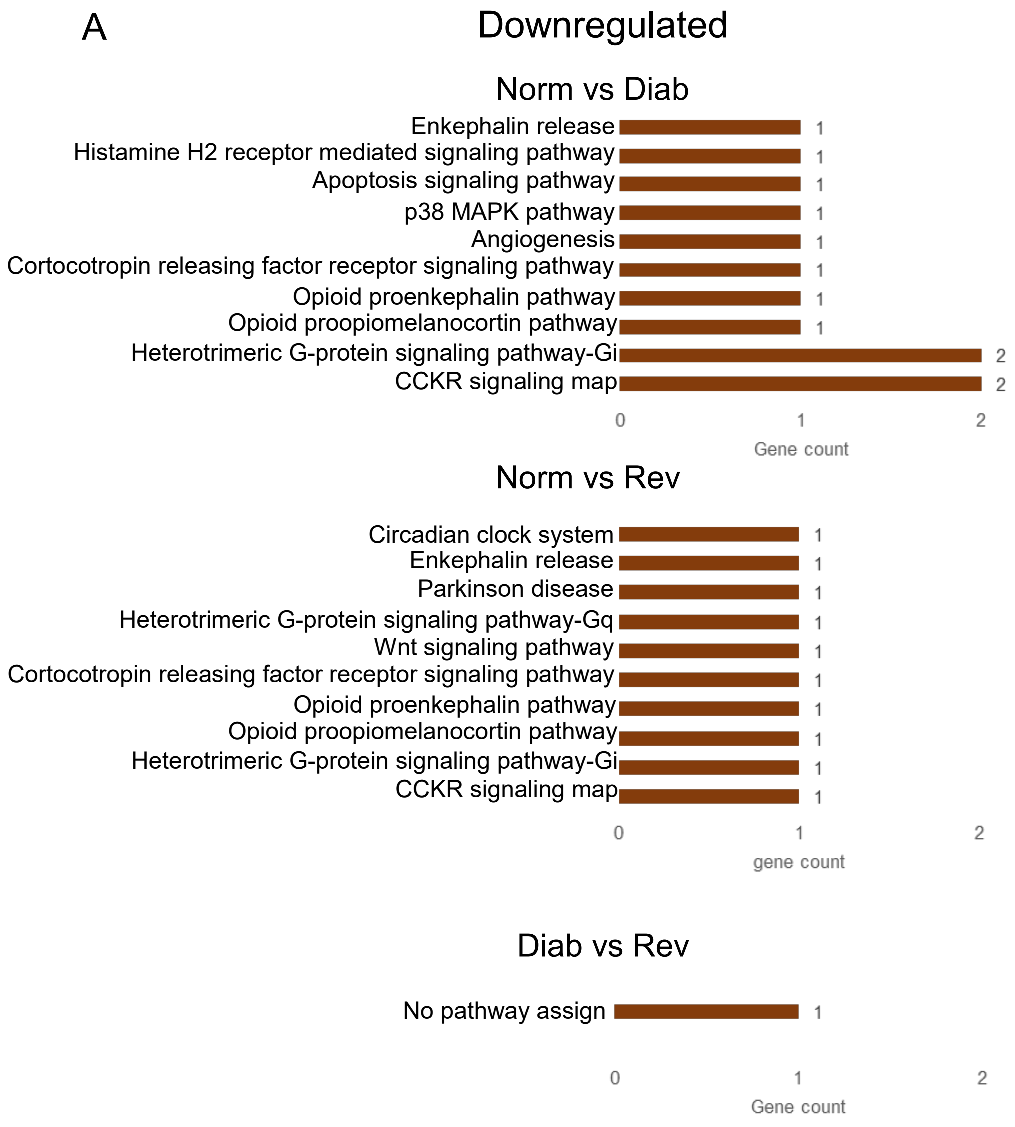

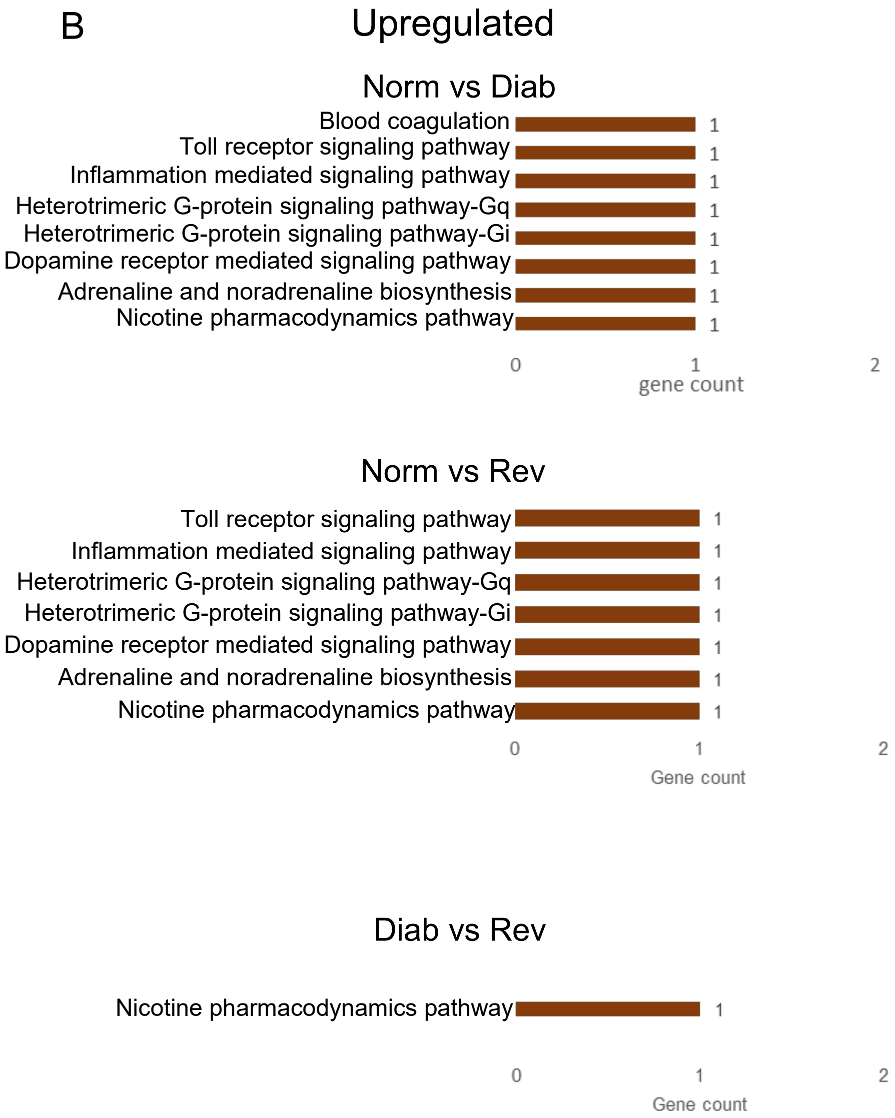
B
Upregulated
Norm vs Diab
Blood coagulation
Toll receptor signaling pathway
Inflammation mediated signaling pathway
Heterotrimeric G-protein signaling pathway-Gq
Heterotrimeric G-protein signaling pathway-Gi
Dopamine receptor mediated signaling pathway
Adrenaline and noradrenaline biosynthesis
Nicotine pharmacodynamics pathway
gene count
Norm vs Rev
Toll receptor signaling pathway
Inflammation mediated signaling pathway
Heterotrimeric G-protein signaling pathway-Gq
Heterotrimeric G-protein signaling pathway-Gi
Dopamine receptor mediated signaling pathway
Adrenaline and noradrenaline biosynthesis
Nicotine pharmacodynamics pathway
Gene count
Diab vs Rev
Nicotine pharmacodynamics pathway
Gene count

Figure S2. LncRNA-mRNA co-expression network.

Co-expression network of 51 differentially expressed retinal LncRNAs and 50 differentially expressed mRNAs, constructed using LncRNA-mRNA pairs with Pearson's correlation coefficient >0.9 and p<0.01, in rats maintained in continuous poor glycemic control for eight months (Diab group). Blue nodes=LncRNAs; Orange nodes= mRNA coding genes; Solid line= Directed, positive correlation and broken line= Undirected, negative correlation.

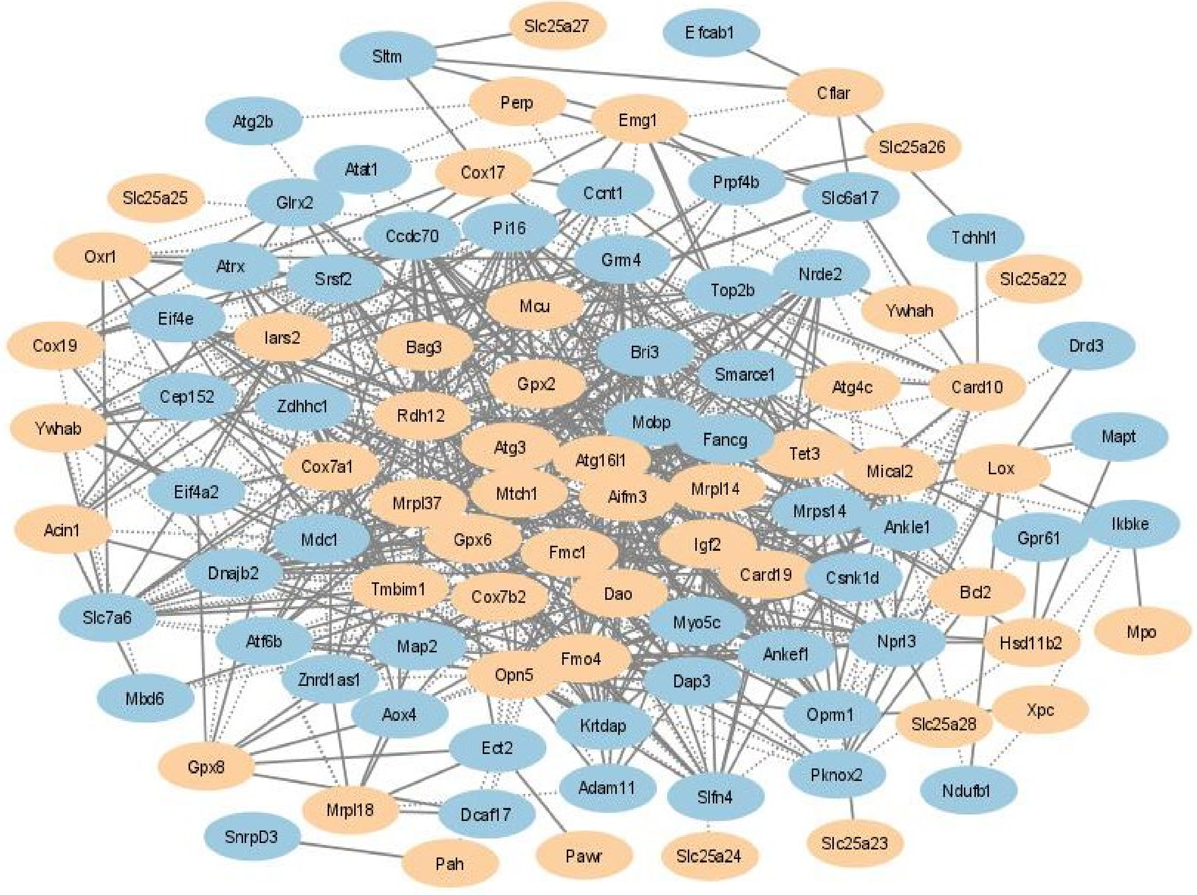

Supplement: Supplementary file 1 — Figure S1. KEGG pathways analysis of differentially expressed LncRNAs. Top KEGG pathways corresponding to (A) downregulated and (B) upregulated retinal LncRNAs in Diab versus Norm and Rev versus Norm groups. Figure S2. LncRNA–mRNA coexpression network. Coexpression network of 51 differentially expressed retinal LncRNAs and 50 differentially expressed mRNAs, constructed using LncRNA–mRNA pairs with Pearson's correlation coefficient >0.9 and p < 0. 01, in rats maintained in continuous poor glycemic control for 8 months (Diab group). Blue nodes = LncRNAs; Orange nodes = mRNA coding genes; Solid line = Directed, positive correlation and broken line = Undirected, negative correlation. [file JDB-16-e70009-s002.pdf]
