# Supplementary material for: Long noncoding RNAs and metabolic memory associated with continued progression of diabetic retinopathy
Source: J Diabetes. 2024 Nov 18;16(11):e70009. doi: 10.1111/1753-0407.70009 (PMC11574110; doi:10.1111/1753-0407.70009)
Supplement: Supplementary file 2 — Table S1. Functional details of LncRNAs. [file JDB-16-e70009-s001.pdf]

Table SI

Functional details of LncRNAs

LncRNA	Transcript	Sequence	Relationship	Regulation	Function of the related genes (Rat Genome Database (mcw.edu))
LOC100362486	XR_347139	CAAAACGATTTATTACTGCAACACAAGCAGGCTACAATACACTTCATACATTTCTTTGGT	intergenic	down	von Willebrand's disease 1 (ortholog); interacts with aflatoxin B1 (ortholog).
Ankef1	XR_591690	TTCGAAAAAAATATGACATCAATCACCCCCGTGATGTTGAACAAAATATTCAGTGTTTCT	exon sense-overlapping	down	Interacts with 1-naphthyl isothiocyanate; 2,3,7,8-Tetrachlorodibenzofuran.
Csnk1d	XR_594964	GGCCCCAGCGGCCTCTGACCGGGCTTCCAGCCACGCAGGCCGCCACCGGCCACAGGGCAG	bidirectional	down	Located in the centrosome and perinuclear region of cytoplasm; cellular response to nerve growth factor stimulus.
Ankle1	XR_341037	ACGTCCCAAGGGTGCTGGACACCATCCTTGGGTCCCCACATCCTCCGCAGGATGCCAGGG	exon sense-overlapping	down	Enables DNA endonuclease; negative regulation of mitotic recombination; hematopoietic progenitor cell differentiation.
Gpr61	XR_351942	TCATAGTCCAGACTAATATTTATACTGAGACAAGGACTGGCCTCCCTGATCTATCTCCTC	exon sense-overlapping	down	G protein-coupled receptor activity and arrestin family protein binding activity.
Nprl3	XR_357451	CGTCGCTCGGTGAAGTCTCGCGAGAGTGAGC	exon sense-overlapping	down	TORC1 signaling and cellular response to

		GCCGTGGTGG AGGCACCCGC TTCCCCGGT			amino acid starvation.
Fancg	XR_346463	TGAAGGCACC GGTGGGACCA GCCTCCACCC ATCGGAAGACC CCTTCGTAACG GTGATCTA	exon sense-overlapping	down	DNA damage response and mitochondrion organization.
LOC100 361265	XR_597622	CTTCCGGTGG CTTTCCCAAGA CCAGTCCTAGA AAGAAGCCAC CCTCAGAAAAG CCATCTC	exon sense-overlapping	down	Orthologous to several human genes including chromosome X open reading frame 49; interacts with clozapine (ortholog) and valproic acid (ortholog).
Ccdc70	XR_596526	CAGCTAAGCAG GTTTGGCCAAC TGACCTGGAC GTGGCCAACA GCCCTGACCT CCAACACA	exon sense-overlapping	down	Located in the plasma membrane. Orthologous to human CCDC70; interacts with bisphenol A; 1,2-dichloroethane (ortholog); 2,2',4,4',5,5'-hexachlorobiphenyl (ortholog).
Tchhl1	XR_145852	GAAGAGAGAG AGGAGTGACA GTCTGAGCTCA AGGACCAAAA GAGGCCCAGG GGAGCTATG	exon sense-overlapping	up	Calcium ion binding activity and transition metal ion binding activity.
Efcab1	XR_595172	TTGACCAAAAT AAGTTTGAGAA ATGCCCTTCAG AGAGAGAGAC GGCTAGGGAA GCAGAAG		up	Calcium ion binding activity and regulation of flagellated sperm motility.
Slc7a6	XR_597221	CTTTGTGAGTG TGCAAGATCCC	exon sense-overlapping	up	L-lysine: L-arginine

		GAGTGTGAGG GGGACTGTGT GTGGGAAAACT GCGAGAT			antiporter activity and arginine binding activity.
Prpf4b	XR_596685	CTGTTTGTCTC CCTGAGGATTA TGGAGAACAAT AACATTTGGGG GTTTAAATAGTA TTTT	exon sense-overlapping	up	Cyclin-dependent protein serine/threonine kinase activity and mRNA cis splicing via spliceosome; regulation of cell cycle.
Srsf2	XR_358012	TAAGAGAATGC TGCGGTCTCCT GTTTGATAAAA GAATATTGGCC AGTATTGCAGA TTTTA	exon sense-overlapping	up	Enables protein kinase C binding activity; involved in response to vitamin E.
LOC100911891	XR_354153	GAGGAGGGAG GACAACCTGTG GATCTTCACTT GATCAACAATG CAGTTGCCAAT GTAATT	intergenic	up	Not available
ENSRNOG00000052421	ENSRNOT00000079148	AGGTGTCTTGC CTCTGTCTCCC GAGTGAGTTTT GACATTAAAGG TGTATCACCAC TAGGT	intergenic	up	Histone-lysine N-methyltransferase SETDB1-like; LOC689873; similar to Histone H3-K9 HMT.
Ikbke	XR_595428	CCGGAATAAGC TAGAAATCCGG GGAGGTGGTT GCTGTAAAGGT CTTCAACTCAG CCAGCT	exon sense-overlapping	up	Ikappa B kinase activity; K48-linked polyubiquitin modification-dependent protein binding activity.
MALAT1	FQ233546	Not available	Intergenic	up	Cellular response to hypoxia; positive regulation of cardiac muscle myoblast

					proliferation; associated with the oxidative stress in the mitochondria; proliferation and metastasis of tumor cells.
NEAT1	Not available	Not available	Intergenic	up	Located in the paraspeckles; acts upstream of, or within, cellular component maintenance; affects mitochondrial dynamics and function
HOTAIR	NR_132646	CCACAGCTGG GAGATTAATCA ACCACACTGAA AATGGGGGGA GGGGAAGGAA GAGTTGGA	intergenic	up	Acts upstream of, or within, hindlimb morphogenesis; post-anal tail morphogenesis; , regulation of histone methylation.
Meg3	NR_131064	Not available	intergenic	down	Chromatin organization; acts upstream of, or within, SMAD protein signal transduction; genomic imprinting.
CARL	NR_131254	Not available	intergenic	up	Located in the cytoplasm; enables pre-miRNA binding activity; acts upstream of, or within, cellular response to anoxia; negative regulation of mitochondrial

					fission; miRNA metabolic process.
CytB	Not available	Not available	antisense	down	Located in the mitochondrial inner membrane; contributes to ubiquinol-cytochrome-c reductase activity; involved in electron transport chain; biomarker of thyrotoxicosis; type 2 diabetes mellitus; maintains mitochondrial genome and stability.
ND5 & ND6	Not available	Not available	antisense	no change	Located in the mitochondrial inner membrane; a part of mitochondrial respiratory chain complex I; enables NADH dehydrogenase activity; involved in response to hydrogen peroxide, hypoxia and organonitrogen compound.
